# Supplementary material for: The Systems Biology Research Tool: evolvable open-source software
Source: BMC Syst Biol. 2008 Jun 29;2:55. doi: 10.1186/1752-0509-2-55 (PMC2446383; doi:10.1186/1752-0509-2-55)
Supplement: Additional file 1 — SBRT Archive. An archive of the current version of the Systems Biology Research Tool. [file 1752-0509-2-55-S1.zip › sbrt-1.4.0/doc/developers_guide/api/sbrt/kernel/fba/comp/InvalidFluxVectorException.html]

InvalidFluxVectorException


|  |  |  |
| --- | --- | --- |
| **Overview** \| **Package** \| **Class** \| **Use** \| **Tree** \| **Deprecated** \| **Index** \| **Help** | |  |
| **PREV CLASS**   **NEXT CLASS** | **FRAMES**    **NO FRAMES**     **All Classes** |  |
| SUMMARY: NESTED \| FIELD \| CONSTR \| METHOD | DETAIL: FIELD \| CONSTR \| METHOD |  |


---


## sbrt.kernel.fba.comp Class InvalidFluxVectorException

```
java.lang.Object
  java.lang.Throwable
      java.lang.Exception
          java.lang.RuntimeException
              sbrt.kernel.fba.comp.InvalidFluxVectorException
```

**All Implemented Interfaces:**: java.io.Serializable

---

``` public class InvalidFluxVectorException extends java.lang.RuntimeException ```

This class is used to indicate that a flux vector is invalid.

**Author:**
:   This class was written and documented by Jeremiah Wright
    while in the Wagner lab.

**See Also:**: Serialized Form

---

**Constructor Summary**

`InvalidFluxVectorException(java.util.Map<java.lang.String,java.lang.Double> fluxVector)`
  
          Constructs a new exception for the provided flux vector.

`InvalidFluxVectorException(java.util.Map<java.lang.String,java.lang.Double> fluxVector,
java.lang.String message)`
  
          Constructs a new exception for the provided flux vector.

`InvalidFluxVectorException(java.util.Map<java.lang.String,java.lang.Double> fluxVector,
java.lang.String message,
java.lang.Throwable cause)`
  
          Constructs a new exception for the provided flux vector.

`InvalidFluxVectorException(java.util.Map<java.lang.String,java.lang.Double> fluxVector,
java.lang.Throwable cause)`
  
          Constructs a new exception for the provided flux vector.


| **Method Summary** | |
| --- | --- |
| `UnmodifiableMap<java.lang.String,java.lang.Double>` | `getFluxVector()`             Returns the invalid flux vector; |

**Methods inherited from class java.lang.Throwable**

`fillInStackTrace, getCause, getLocalizedMessage, getMessage, getStackTrace, initCause, printStackTrace, printStackTrace, printStackTrace, setStackTrace, toString`

**Methods inherited from class java.lang.Object**

`clone, equals, finalize, getClass, hashCode, notify, notifyAll, wait, wait, wait`

**Constructor Detail**

### InvalidFluxVectorException

```
public InvalidFluxVectorException(java.util.Map<java.lang.String,java.lang.Double> fluxVector)
```

:   Constructs a new exception for the provided flux vector.

    **Parameters:**: `fluxVector` - the invalid flux vector.

---


### InvalidFluxVectorException

```
public InvalidFluxVectorException(java.util.Map<java.lang.String,java.lang.Double> fluxVector,
                                  java.lang.String message)
```

:   Constructs a new exception for the provided flux vector.

    **Parameters:**: `fluxVector` - the invalid flux vector.: `message` - a message useful for debugging.

---


### InvalidFluxVectorException

```
public InvalidFluxVectorException(java.util.Map<java.lang.String,java.lang.Double> fluxVector,
                                  java.lang.Throwable cause)
```

:   Constructs a new exception for the provided flux vector.

    **Parameters:**: `fluxVector` - the invalid flux vector.: `cause` - the exception used to initially indicate that the flux vector is invalid.

---


### InvalidFluxVectorException

```
public InvalidFluxVectorException(java.util.Map<java.lang.String,java.lang.Double> fluxVector,
                                  java.lang.String message,
                                  java.lang.Throwable cause)
```

:   Constructs a new exception for the provided flux vector.

    **Parameters:**: `fluxVector` - the invalid flux vector.: `message` - a message useful for debugging.: `cause` - the exception used to initially indicate that the flux vector is invalid.


**Method Detail**

### getFluxVector

```
public UnmodifiableMap<java.lang.String,java.lang.Double> getFluxVector()
```

:   Returns the invalid flux vector;

    :   **Returns:**: the invalid flux vector;


---


|  |  |  |
| --- | --- | --- |
| **Overview** \| **Package** \| **Class** \| **Use** \| **Tree** \| **Deprecated** \| **Index** \| **Help** | |  |
| **PREV CLASS**   **NEXT CLASS** | **FRAMES**    **NO FRAMES**     **All Classes** |  |
| SUMMARY: NESTED \| FIELD \| CONSTR \| METHOD | DETAIL: FIELD \| CONSTR \| METHOD |  |


---
